# Effectiveness of Vitamin D Supplementation in the Management of Multiple Sclerosis: A Systematic Review

**DOI:** 10.3390/ijms20061301

**Published:** 2019-03-14

**Authors:** Monika Berezowska, Shelly Coe, Helen Dawes

**Affiliations:** Centre for Movement, Occupational and Rehabilitation Sciences, Oxford Brookes University, Oxford OX3 0BP, UK; mberezowska84@gmail.com (M.B.); hdawes@brookes.ac.uk (H.D.)

**Keywords:** Vitamin D, Multiple Sclerosis, symptom

## Abstract

Objective: to examine the extent of effect vitamin D in Multiple Sclerosis (MS) on pathology and symptoms. Methods: A literature search was performed in November 2018 (CRD42018103615). Eligibility criteria: randomised control trials in English from 2012 to 2018; a clinical diagnosis of MS; interventions containing vitamin D supplementation (vitamin D3 or calcitriol) in disease activity compared to a control/placebo; improvement in: serum 25(OH)D, relapse rates, disability status by Expanded Disability Status Scale (EDSS) scores, cytokine profile, quality of life, mobility, T2 lesion load and new T2 or T1 Gd enhancing lesions, safety and adverse effects. Risk of bias was evaluated. Results: Ten studies were selected. The study size ranged from 40 to 94 people. All studies evaluated the use of vitamin D supplementation (ranging from 10 to 98,000 IU), comparing to a placebo or low dose vitamin D. The duration of the intervention ranged from 12 to 96 weeks. One trial found a significant effect on EDSS score, three demonstrated a significant change in serum cytokines level, one found benefits to current enhancing lesions and three studies evaluating the safety and tolerability of vitamin D reported no serious adverse events. Disease measures improved to a greater extent overall in those with lower baseline serum 25(OH)D levels. Conclusions: As shown in 3 out of 10 studies, improvement in disease measures may be more apparent in those with lower baseline vitamin D levels.

## 1. Introduction

There is increasing evidence suggesting that specific environmental factors, such as exposure to infectious agents, smoking, poor diet and inadequate levels of vitamin D can influence the disease course of multiple sclerosis (MS) [1]. Adequate vitamin D status is documented as associated with reduced prevalence, activity and progression of disease in MS, and therefore high intake of vitamin D may be a useful addition to standard treatment [2]. Numerous observational studies investigating variations in sunlight exposure, latitude and diet have supported the correlation between a high serum concentration of vitamin D and reduced severity of the disease course in established MS [3,4].

Epidemiologic and experimental studies investigating the effectiveness of vitamin D supplementation in MS have shown that low serum vitamin D levels may exacerbate MS symptoms and therefore are associated with higher relapse rates, new lesions, and greater degree of disability [5,6,7,8,9]. Although there has been much research performed into the role of vitamin D in MS risk and progression, due to heterogeneity of study designs, there have been conflicting results. For example, baseline serum 25(OH)D levels often differ between studies. Reviews on the topic have thus far been inconclusive and are mainly focused on the role of vitamin D and risk of developing MS, rather than the outcomes after diagnosis [7]. The only two other systematic reviews to date on vitamin D for the clinical efficiency of MS did not use the full range of terms for vitamin D nor was bias assessed [10] and didn’t assess cytokine outcomes nor looked at the effects of baseline Vitamin D levels on outcomes [11]. The aim of this review is to assess the evidence from existing randomised controlled trials for the clinical effectiveness of vitamin D supplementation compared to placebo supplementation in the disease and symptom management of people with MS as measured by: improvement in: serum 25(OH)D, relapse rates, disability status by Expanded Disability Status Scale (EDSS) scores, cytokine profile, quality of life, mobility, T2 lesion load and new T2 or T1 Gd enhancing lesions, safety, and adverse effects.

## 2. Methods

The systematic review was registered in PROSPERO (CRD42018103615). A literature search was performed in November 2018. Table 1 shows the search terms and number of hits for each database. Reference lists were hand searched for additional papers. Twenty percent of abstracts and papers were checked by a second reviewer.

Studies were included if they met each of the following criteria: A clinical diagnosis of MS; Direct relevance of vitamin D supplementation on the management of MS compared to a low dose vitamin D or a placebo supplement; Primary outcome measurements in one or more of: serum 25(OH)D, relapse rates, disability status by EDSS scores, cytokine profile, quality of life, mobility, T2 lesion load and new T2 or T1 Gd enhancing lesions, safety and adverse effects; Randomised control trial (RCT) with a control and intervention group; Published from 2012 and in English; The published data available in full text; Only human randomised controlled clinical trials.

**Figure 1:**
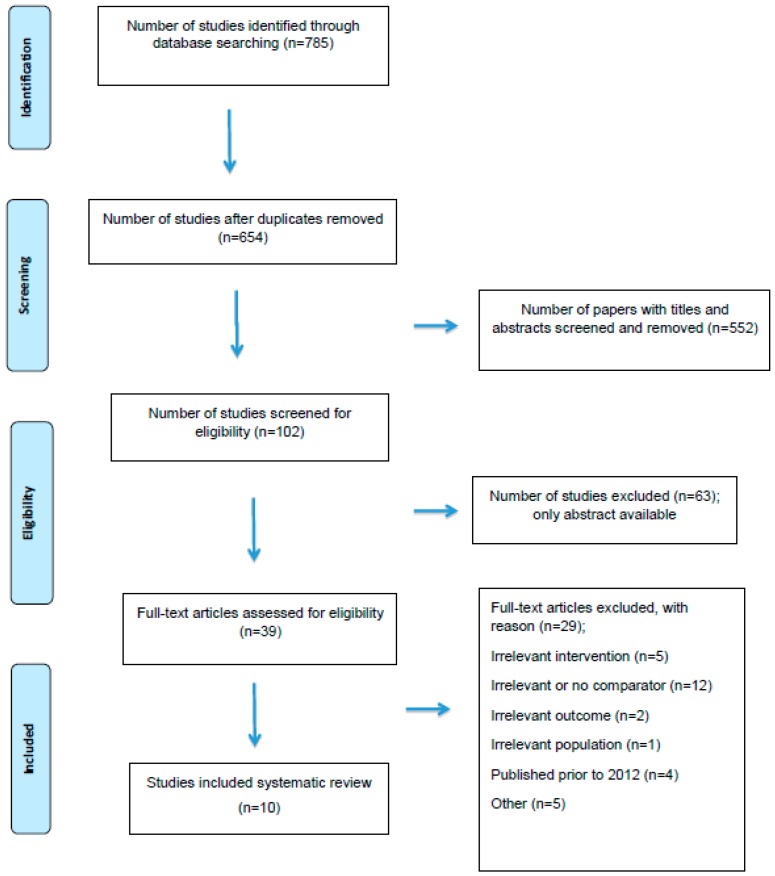
Preferred Reporting Items for Systematic Reviews and Meta-Analyses (PRISMA) flow diagram reporting the number of records identified, included and exclude through the different phases of a systematic review.

The Preferred Reporting Items for Systematic Reviews and Meta-Analyses (PRISMA) guidelines were followed and the flow diagram is presented in Figure 1. Bias was assessed using the RoB 2.0 tool at a study level. Data were extracted by one reviewer, and a selection of excluded abstracts and all full papers, and included papers were confirmed by a second reviewer.

## 3. Results

Out of 785 studies, ten RCTs were identified as eligible for this review after the application of the inclusion and exclusion criteria. The information from each selected study was extracted, and detailed characteristics are shown in Table 2.

### 3.1. Bias

All studies were considered to have a low risk of bias and therefore systematic error was unlikely and there was no threat to validity.

### 3.2. General Characteristics

The studies reviewed in this report were all double-blind RCTs that focused on the role of vitamin D supplementation in the management of people with MS. Country of origin is shown in Table 2. Inclusion and exclusion criteria, in addition to other demographic information is shown in Table 3. 

### 3.3. Participants

The studies size ranged from 40 to 94 people with MS. In these ten studies, there was a total of 627 adult participants (463 female and 164 male). Food intake of vitamin D and smoking status were not reported in any of the RCTs.

### 3.4. Study Objectives

Seven studies looked at the effect of vitamin D on immunological and inflammatory measures [12,13,14,15,16,17,18]. Outcomes related to functional ability were assessed in two studies [19,20] and relapse rate was assessed in four studies [12,19,20,21]. Disability and/or disease progression was assessed in five studies [12,18,19,20,21] and safety and tolerability of vitamin D supplementation was sought in four [12,13,20,21]. The studies by [16] and [18], by [15] and [19] and by [14] and [20] were based on the same trial however measured different outcomes and as such were treated in this review as separate studies.

### 3.5. Interventions

The intervention dose varied across studies. All studies evaluated the use of vitamin D supplements of various doses (ranging from 10 to 98,000 IU), frequency (usually delivered weekly) and formulation (vitamin D3 and calcitriol). Most studies (*n* = 9) reported concomitant immunomodulatory therapy, often interferon-β as well as different requirements relating to vitamin D and calcium supplementation that were used at baseline. The duration of the vitamin D interventions varied between studies, ranging from 12 to 96 weeks. The included studies (*n* = 8) compared vitamin D supplementation (321 participants) to placebo (264 participants) or versus low dose of vitamin D (*n* = 2; 42 participants). A variety of clinical and biochemical outcome measures were assessed at the baseline and the end of the study for intervention and control groups.

### 3.6. Serum 25(OH)D Levels

Nine of ten studies in this review measured the serum 25(OH)D concentration for both intervention and control group (low dose vitamin D) as an outcome parameter (Table 4). Across studies, mean improvements in cytokine profile or EDSS were seen for those with low baseline plasma Vitamin D levels (*n* = 3). Key findings and significance are shown in Table 5.

### 3.7. Immunologic Markers

Seven of the ten studies in the review used change in serum cytokines level as an outcome parameter with mixed results found and large heterogeneity in markers assessed across studies. Best support was found for Ashtari et al. [16] and Sotirchos et al. [13] in which significant benefits were seen in the high dose Vitamin D groups on IL-10, and on IL-17+CD4+T and CD4+T cells these were also the studies where baseline vitamin D levels were lower than normal.

Golan et al. [12] reported a significant increase in serum IL-17 concentration in people allocated to the low-dose vitamin D from a mean of 4.01 to 9.14 pg/mL at 48 weeks (*p* = 0.037) and a heterogeneous IL-17 response was observed in the high-dose vitamin D group. Therefore, there was a decrease and thus a beneficial change in 40% of participants and increase and negative change in 45% of participants after 3 months while 15% had IL-17 levels below the detection threshold at both time points. Aivo et al. [14] detected a significant increase in LAP (TGF-β) levels in the vitamin D arm after 48 weeks from a mean of 47 to 55 pg/mL (*p* = 0.02), while in those receiving placebo, this level increased but these changes were not significant (*p* = 0.173). Moreover, no significant difference in other cytokines concentration was reported in either group. Ashtari et al. [16] found that serum IL-10 concentration changed significantly in people receiving vitamin D for 12 weeks (*p* = 0.015) from a median of 12.58 to 13.76 pg/mL. Rosjo et al. [15] indicated no significant differences from baseline values for any of the inflammation markers between those receiving vitamin D or placebo after 96 weeks of treatment. Additionally, people with MS on immunomodulatory treatment (mostly consisting of IFN-b) were observed to have higher mean baseline levels of inflammation markers (IL-1Ra and CXCL16) compared to those not on therapy. However, there was no clear correlation between vitamin D supplementation and immunomodulatory treatment and its influence on the inflammation markers. Toghianifar et al. [18] showed that the proportion of cells including: nTreg, iTreg, Breg, IL4+ Th cells, IL5, and LAP (TGFβ) was not affected by a high-dose vitamin D supplementation. No difference in IL-17 levels between those who received vitamin D, and those who received placebo were observed at 12 weeks. Muris et al. [17] found no beneficial effects of a high-dose vitamin D supplementation on the circulating regulatory immune cell compartment (the fraction of Treg as the proportion of CD4+ T cells, nTregs, IL10+ Th cells) in those with MS. Sotirchos et al. [13] detected a significant change in the proportion of proinflammatory IL-17+CD4+T cells in the high-dose group (*p* = 0.016) from a mean of 9.32% to 5.62%, while no difference was observed in the low-dose group (*p* = 0.53). Moreover, a significant difference in IL-17+CD4+T cells in the high-dose group versus low-dose group was reported (*p* = 0.039). Greater reduction in the proportion of IFN-γ+CD4+ T cells and IFN-γ+IL-17+CD4+ T cells was noted in the high-dose group versus the low-dose group but did not reach statistical significance (*p* = 0,12; *p* = 0.14). Also, a decreased proportion of effector memory CD4+T cell was noted after high-dose vitamin D supplementation from a mean of 40.56% to 30.69% (*p* = 0.021). The proportion of central memory and naive CD4+T cells increased significantly (*p* = 0.019; *p* = 0.043) in the high-dose group from a mean of 50.07% to 60.96% and from 38.94% to 42.2%, respectively.

### 3.8. Functional Measures

Only one study assessed functional measures and although there were trends for improvements in the Vitamin D groups, there were no statistically significant changes between the intervention and placebo groups. Soilu-Hänninen et al. [20] demonstrated that vitamin D supplementation resulted in fewer new T2 lesions (a mean of 0.5 compared to a mean of 1.1 in the placebo group). However, the difference between vitamin D and placebo groups was not statistically significantly different (*p* = 0.286). Participants assigned to vitamin D demonstrated lower total number of T1 Gd enhancing lesions (0.6 to 0.1) while in the placebo group no change was reported and a higher decrease in T1 enhancing lesion volume in the vitamin D group (from 57 mm^3^ to 3.1 mm^3^) compared with the placebo group (from 62 mm^3^ to 29 mm^3^) but again the difference between the treatment groups was not statistically significant (*p* = 0.004, *p* = 0.320, respectively). There were no statistically significant differences between the treatment groups in timed 10 foot tandem walk (TTW10; *p* = 0.076) (change from a mean of 11.7 to 9.7 in the vitamin D group and from 9.6 to 11.2 in the placebo group) and T25FW (*p* = 0.932) at the end of the study (change from a mean of 6.0 to 5.3 in the vitamin D group and from 4.7 to 5.1 in the placebo group).

### 3.9. Relapse Rate

Four of ten studies in this review investigated the effect of supplementation with vitamin D on relapse rates, with no significant differences between the vitamin D and control groups. Kampman et al. [19] demonstrated that vitamin D supplementation resulted in an increase in annualised relapse rate (ARR, calculated as the total number of relapses experienced divided by the sum of participants and duration of follow-up) from 0.11 to 0.14, whereas in placebo group a decrease from 0.15 to 0.8 was reported. The difference between vitamin D and placebo group after 96 weeks was not significant (*p* = 0.25). Shaygannejad et al. [21] documented that the relapse rate decreased significantly after 48 weeks from a mean of 1.04 to 0.32 in people who received vitamin D (*p* < 0.001) and from 1.04 to 0.40 in those who received placebo (*p* < 0.001). The study by Golan et al. [12] found an increase in ARR in patients with MS following the treatment with high-dose per day from 0.28 to 0.51 and decrease in the low-dose from 0.38 to 0.34 at week 48, but this difference was not statistically significant (*p* = 0.32). The study by Soilu-Hänninen et al. [20] found a decrease in ARR in both treatment arms: in people who received vitamin D from a mean of 0.49 to 0.26 and from 0.51 to 0.28 in those who received placebo, yet with no significant difference between groups.

### 3.10. Disability

Five of ten studies reported EDSS score as outcome parameter with only one showing a benefit after supplementation with vitamin D. Kampman et al. [19] noted that EDSS score did not differ significantly between the vitamin D and placebo group after 96 weeks (*p* = 0.97). Shaygannejad et al. [21] found that EDSS score increased significantly (*p* < 0.01) in a placebo group from a mean of 1.7 to 1.94, whereas it did not change in people receiving vitamin D and therefore there was no significant difference in scores at the end of the trial between intervention and control groups (*p* > 0.05). Also, Golan et al. [12] demonstrated that high-dose vitamin D supplementation was not associated with reduced disability score with no significant change in EDSS score between two groups (*p* = 0.26). In contrast, Toghianifar et al. [18] showed a significant difference in EDSS scores between people allocated to vitamin D group (supplemented with 50,000 IU every five days) and placebo group after 12 weeks (*p* = 0.033) in favour of the vitamin D group, and the baseline vitamin D levels in the participants from this study was below the minimum recommendation. Soilu-Hänninen et al. [20] found no significant change in EDSS score between two groups (*p* = 0.071).

### 3.11. Safety and Tolerability

Four of the ten studies in this review determined the effect of high-dose vitamin D supplementation among people with MS in terms of safety and tolerability and none of the studies reported significant differences between control/placebo and vitamin D groups nor were any of the adverse events serious in either group. Shaygannejad et al. [21] showed that vitamin D treatment use up to 0.5 μg/day of calcitriol appeared to be safe and well tolerated by those with MS. The adverse events noted were mild in severity. The most frequently reported included constipation (*n* = 6 and *n* = 4), dyspepsia (*n* = 6 and *n* = 2), fatigue (*n* = 4 and *n* = 5), and headache (*n* = 2 and *n* = 1) in vitamin D and placebo groups, respectively. There were no significant differences in frequency of events between people who received vitamin D and those who received placebo. Golan et al. [12] indicated that a dose of 4370 IU/day over a 48-week period was safe in people with MS. There were no instances of hypercalcemia and no reports on new adverse events that could be vitamin D supplement related. Sotirchos et al. [13] found that a dose of 10,400 IU of cholecalciferol per day for 24 weeks was safe and tolerable in people with MS, with no serious adverse events. Soilu-Hänninen et al. [20] found no significant differences between the treatment arms in any of the other clinical chemistry parameters studied. No dose adjustments were necessary. Lack of MxA response (MxA < 50 mg/L) was detected in three people in both treatment arms at 12 months. Diarrhoea was a side effect in (*n* = 5 and *n* = 2) and fever was noted (*n* = 2 and *n* = 5) in the vitamin D group and placebo group, respectively. All other adverse events occurred in a similar number of participants in both groups. There was one serious adverse event in the vitamin D group (erysipelas in the interferon injection site treated with intravenous antibiotics in hospital) and two in the placebo group (elective hip surgery and elbow fracture).

## 4. Discussion

This review found some evidence for benefits of vitamin D supplementation, specifically for those with serum levels at the lower normal range in people with RRMS. Therefore, baseline serum vitamin D levels may be a predictor of improvements in disease pathology from vitamin D supplementation, cytokine profile and disability status, but possibly also relapse rate, quality of life, mobility, T2 lesions load and new T2 and T1 Gd enhancing lesions. Five out of ten studies showed improvement in: ARR(x2), EDSS(x2), IFN-gamma, IL-17A, IL-9, IL 10, 17+CD4+ T cells, CD161+CD4+ T cells, and effector memory CD4+ T cells, the proportion of central memory CD4+ T cells and naive CD4+ T, TTW10, T25FW, and MRI brain lesion markers, and these were shown in the intervention group compared with the control/placebo group. Another similar review to date differed in that cytokine outcomes were not assessed and the effects of baseline Vitamin D levels on outcome measures was not explored [11]. McLaughlin et al. [11] found that in higher dose vitamin D arms, there were actually adverse changes in ARR and EDSS and therefore although supplementation may have beneficial effects, there may be specific doses that should be considered. Jagannath et al. [22] looked at outcome measures including fatigue and HRQOL yet found conflicting results in part due to the heterogeneity of the study designs and different doses used. Zheng et al. [23] only looked at changes in ARR and EDSS score, with no beneficial effect of vitamin D as an add-on therapy on either outcome. Whilst further research is needed, this review highlights that all studies on the topic should include baseline vitamin D as part of the assessment. There was also low risk of adverse effects and low risk of bias for all studies and therefore the validity can be considered high. This review not only includes a more extensive search strategy and evaluates bias and although some of the included studies between reviews are similar, the current review is more up to date and encompasses a wider range of symptoms and pathology in MS.

The present consensus on the use of vitamin D supplementation in the management of MS is based on the hypothesis that the serum 25(OH)D is associated with prevalence and severity of the disease course in established MS. Therefore, its measurements are undertaken as part of the clinical management of MS in order to detect vitamin D insufficiency, correct it with supplementation at recommended doses and achieve the beneficial immunological effects [4]. All but one study assessed levels of serum 25(OH) and all reported a significant increase in 25(OH)D levels following vitamin D supplementation. However, the increase in 25(OH)D levels did not appear to affect all MS-related outcomes in the reviewed studies. If participants had 25(OH-D) levels at the lower end of normal at baseline, a high dose vitamin D supplement intervention may contribute to bettering of physiological mechanisms and resulting symptoms, yet if baseline levels are at the higher end of normal (i.e., 50~nmol/L) then further benefits may not be experienced. In the study by Ashtari et al. [16] and Sotirchos et al. [13] participants had levels towards the lower end of normal thereby possibly resulting in the resulting significant benefit in IL-10 and a variety of mechanistic improvements, respectively. Toghianifar et al. [18] found a resulting improvement in EDSS score which wasn’t seen in other studies in this review, and again the participants in this study had baseline 25(OH-D) levels at the lower end of normal. All other studies had participants with higher baseline levels and also contained more varied results, with fewer significant changes between groups.

When looking at the immunological outcomes, the reviewed studies reported mixed effects of vitamin D supplementation. Vitamin D plays an important role in immune system function by reducing the production of proinflammatory cytokines and inducing the production of anti-inflammatory cytokines [24]. Only two selected studies detected a significant increase in levels of anti-inflammatory cytokines in the vitamin D group and therefore findings of studies evaluating the effect of the vitamin D supplementation on the reduction of proinflammatory cytokines are conflicting. The heterogeneity of intervention effects on immunologic activity reported in reviewed trials may be explained by considering possible confounding parameters including dosage and duration of administering vitamin D supplementation and supports previous findings demonstrating that a more pronounced immunologic impact of vitamin D supplementation was reported in vitamin D doses up to 40,000 IU per day [24]. Moreover, the fact that almost all participants in above trials were treated by immunomodulatory treatment, which mostly comprised interferon-beta (IFN-β) therapy (Table 6), may have altered the cytokine responses to vitamin D and/ or made it more difficult to determine the isolated effect of vitamin D supplementation and therefore beneficial effects of an increase in 25(OH)D on the outcome markers examined may be undetectable due to the strong immunomodulatory effect of IFN-β [25]. It has been suggested that type of therapy a person receives may influence the observed impact of vitamin D supplementation [26]. Notwithstanding, some studies demonstrated a synergistic immunomodulatory effect of IFN-β and vitamin D that induce favourable alterations in the inflammatory profile in people with MS [12,13]. Also, when considering the study conducted by Golan et al. [12] and Sotirchos et al. [13] including low-dose of vitamin D as a comparator may reduce the ability to notice minor differences compared to the use of a placebo. Although results of studies evaluating changes in immunological profiles in people with MS are not consistent, they suggest that supplementation of vitamin D promotes the immune regulatory cytokines and reduces proinflammatory immune parameters. Only two studies assessed changes in functional measures, and although the relationship between vitamin D and improved outcomes in participants with MS was found by Soilu-Hänninen et al. [20] T1 enhancing lesions and trends in MRI burden of disease (BOD) and EDSS, there is currently not enough clinical data to suggest the effectiveness of the treatment.

The correlation between 25(OH)D and reduced relapse rates have been found in several prospective cohort studies. The study by Laursen et al. [27] reported that the increase in serum 25(OH)D level was associated with decreases in ARR in those with RRMS. Those results were in line with a previously conducted cohort study by Simpson et al. [28] investigating a role of 25(OH)D levels in modulating MS clinical course in 145 participants with RRMS that suggests a benefit of serum 25(OH)D level on relapse rates at levels approximately 100 nmol/L. However, three reviewed studies evaluating vitamin D supplementation in management of MS have demonstrated no effect of 25(OH)D on relapse rate. Although mean serum 25(OH)D level more than doubled in the high-dose intervention groups in the study by Kampman et al. [19], Soilu-Hänninen et al. [20] and Golan et al. [12], they found no significant difference in ARR between groups at the end of the study period (96 and 48 weeks, respectively). Also, Shaygannejad et al. [21] failed to detect significant difference in relapse rate between the intervention and control groups at 48 weeks although the relapse rate decreased significantly in the vitamin D group. One possible explanation for the discrepancies between findings of above trials and previous studies may be related to eligibility criteria for included participants, vitamin D dosage and form, and duration of the intervention. Other explanations for the results in these RCTs may be related to the low ARR at baseline which could contribute to the absence of significant effects. In addition, the study conducted by Kampman et al. [19] enabled participants to continue the use of vitamin D supplements they used prior the study, which contributed to comparatively high 25(OH)D concentration in the placebo group and a difference between groups could not be detected.

High levels of 25(OH)D (>50 nmol/L) have also been shown to be associated with reduced disability measured by EDSS in MS [29]. Based on the evidence contained in this review, the effect of vitamin D supplementation on reducing disability remains unclear. Kampman et al. [19], Soilu-Hänninen et al. [20], Shaygannejad et al. [21] and Golan et al. [12] reported no significant change in EDSS score between the intervention and control groups. Conversely, a trial conducted by Toghianifar et al. [18] demonstrated a significant positive difference in EDSS scores between participants allocated to vitamin D vs placebo groups. Although the inclusion criteria were limited to participants with EDSS < 4 that indicate absence of observations in the higher EDSS range, a dose of 50,000 IU vitamin D every five days after 12 weeks was associated with less neurological disability.

Additionally, four studies looked at the safety and tolerability of high dosing regimens of vitamin D supplementation through the duration of the intervention. Through the studies observed it could be clearly recognised that vitamin D treatments were relatively safe, well-tolerated, and no concerning adverse events such as hypercalcemia and hypercalciuria triggered by high doses of vitamin D were reported. This is consistent with findings from previous studies that demonstrated safety of high-dose vitamin D below the daily limit of 10,000 IU in MS [30]. All other adverse events occurred in a similar number of participants in both groups for all studies. There was one serious adverse event in the vitamin D group (erysipelas in the interferon injection site treated with intravenous antibiotics in hospital) and two in the placebo group (elective hip surgery and elbow fracture). What can be concluded from this systematic review is that it seems participants in all studies adhered to the vitamin D interventions due to a resulting increase in serum levels in all studies (*n* = 9), and therefore the safety and tolerability of supplementation at high doses can be considered a reliable outcome.

## 5. Limitations

As the reviewed studies took place in different geographic locations, sun exposure was different amongst groups and makes the comparison less reliable. Of note, all studies recruited participants with RRMS in order to ensure the homogeneity of the treatment groups in terms of the disease course and mechanisms. However, it has been demonstrated that immunomodulatory strategies employed for RRMS are not considered effective when applied in PPMS, suggesting cause for caution when generalising results to the greater MS population. Disease duration before the commencement of treatment varied between 4 months to 27 years and the time at which vitamin D intervention is implemented may affect the effectiveness of the treatment. Some studies assessed clinical endpoints such as relapse rates, disability scores, and physical changes, while some assessed only biomarker outcomes. As a result, heterogeneity of outcomes may have affected end-line comparisons and made doing a meta-analysis unfeasible.

## 6. Conclusions

Vitamin D supplementation may be a promising treatment and represents a reliable background for further exploration of potential benefit for MS regarding clinical improvements. A high dose vitamin D supplement intervention may contribute to bettering of physiological mechanisms if baseline plasma levels are at the lower end of normal. Further research addressing the matters discussed above is required before a causal association between vitamin D supplementation and disease activity in people with MS can be established.

## Figures and Tables

**Table 1:** Key search databases and search terms.

Database Searched	Search Terms Used	Number of Results	Date of Search
PubMed	“Multiple Sclerosis” or “MS” AND “vitamin D supplementation” OR “vitamin D” OR “cholecalciferol” OR “ergocalciferol” OR “calcitrol”	215	01/11/2017
Web of Science	• As above	197	04/11/2017
CINAHL	• As above	19	12/11/2017
Science Direct	• As above	354	12/11/2017
Total		785	

**Table 2:** Characteristics of selected studies.

Reference	Participant Demographics	Study Design, Duration and Country of Origin	Intervention	Outcome Measures
[19]	68 participants (48f, 20m) with MS; Age mean (range) in vitamin D group 40 (21–50) and placebo 41 (26–50); BMI in vitamin D group 28 and placebo group 26	Double-blind placebo- controlled RCT; 96 weeks; Norway	35 participants received supplementation with 20,000 IU vitamin D3 (cholecalciferol)per week; comparator 33 participants received placebo	Serum levels of 25(OH)D; ARR; EDSS; MSFC components; grip strength; FSS
[21]	50 participants (424f, 6m) with RRMS; Age mean (SD) in vitamin D 38.6 (8.4) and placebo 37.9 (7.9); No BMI	Double-blind placebo-controlled RCT; 48 weeks; Iran	25 participants received 0.25 μg/d of calcitriol for 2 weeks and then 0.5 μg/d; comparator 25 participants received placebo	EDSS; relapse rate
[12]	45 participants (32f, 13m) with RRMS; Age mean in high-dose group 43.1 (21.7–63.7) and in low-dose group 43.6 (26.7–63.9); No BMI	Double-blind placebo-controlled RCT; 48 weeks; Israel	High-dose group, 24 participants received 75,000IU vitamin D3 solution every 3 weeks in addition to 800 IU vitamin D3 per day (total 4370 IU); comparator low dose group, 21 participants received placebo every 3 weeks in addition to 800 IU/d of vitamin D3	Serum levels of 25(OH)D; FLS; serum calcium, PTH, cytokine levels (IL-17, IL-10, and IFN-γ); EDSS, relapses, adverse events; QoL
[14]	59 participants (37f, 22m) with RRMS; Age mean (range) in vitamin D 38 (22–53) and in placebo 35 (24–53); BMI 24 kg/m^2^	Double-blind RCT; 48 weeks; Finland	30 participants received 20,000 IU of vitamin D3 (cholecalciferol) per week; comparator 29 participants received placebo	Serum levels of 25(OH)D; inflammatory cytokine: Serum concentrations of LAP (TGF-β); IFN-γ, IL-17A, IL-2, IL-10, IL-9, IL-22, IL-6, IL-13, IL-4, IL-5, IL-1β and TNF-α
[16]	89 participants (75f, 14m) with RRMS; Age mean (SD) in vitamin D group 31.50 (7.60) and placebo 34.60 (10.12); No BMI	Double-blind placebo-controlled RCT; 12 weeks; Iran	High-dose vitamin D group, 44 participants received 50,000 IU of vitamin D3 every 5 days; comparator 45 participants received placebo	Serum levels of 25(OH)D; serum calcium; serum interleukin 10 (IL-10) levels
[15]	68 participants (48f, 20m) with RRMS; Age mean (range) in vitamin D group 40 (21–50) and placebo 41 (28–50); BMI vitamin D group 25.9 and placebo 26.5	Double-blind placebo-controlled RCT; 96 weeks; Norway	36 participants received 20,000 IU vitamin D3 per week; comparator 32 participants received placebo	Serum 25(OH)D; 11 serum markers of inflammation, bone mineral density, clinical disease activity, disease progression: ALCAMd, CCL21e, CXCL16f, IL-1Rag, MMP-9h, OPGi, OPNj, PTX3k, sFRP3l, sTNF-R1m, TGF-b1n
[18]	89 participants (75f, 14m) with RRMS; Age mean (SD) in vitamin D group 31.50 (7.60) and placebo 34.60 (10.12); No BMI	Double-blind placebo-controlled RCT; 12 weeks; Iran	44 participants received oral vitamin D3 50,000 IU every 5 days; comparator 45 participants received placebo	Serum levels of 25(OH)D, serum calcium, IL-17
[17]	53 participants (35f, 18m) with RRMS; Age mean (SD) in vitamin D group 37.7 (7.2) and placebo 37.2 (9.6); BMI ≥ 25 kg/m^2^	Double-blind placebo-controlled RCT; 48 weeks; Netherlands	30 participants received high-dose vitamin D3 supplementation 7000 IU/d for 4 weeks, followed by 14,000 IU/d; comparator 23 participants received placebo	Serum 25(OH)D; serum interleukin 10 (IL-10) levels; cytokine expression of IL4, IFNγ, IL17, IL22, GMCSF and TNFα by CD3+ CD8− T lymphocytes
[13]	40 participants (28f, 12m) with RRMS; Age mean (SD) in high-dose group 41.3 (8.1) and placebo 38.8 (8.8); No BMI	Double-blind RCT; 24 weeks; United States	High-dose group, 19 participants received 10,000 IU/d of cholecalciferol; comparator low-dose group, 21 participants received 400 IU/d of cholecalciferol	Serum 25(OH)D levels; adverse events, relapses, IFN-γ+ IL-17+ CD4+ T cells
[20]	66 participants (41f, 15m) with RRMS; Age median (range) in vitamin D group 39 (22–53) and placebo 35 (24–53); BMI median (range) in vitamin D group 24 (18–40) and placebo 24 (19–38)	Double-blind placebo controlled RCT; 48 weeks; Finland	34 participants received oral vitamin D3 (cholecalciferol) 20,000 IU once a week; comparator group 32 participants received placebo	Serum levels of 25(OH)D; PTH level, T2 BOD; total number of Gd enhancing T1 lesions; new/enlarging T2 lesions; Gd enhancing lesion volume; MRI activity; ARR, EDSS, T25FW and TTW10

ARR, annualised relapse rate; EDSS, Expanded Disability Status Scale; MSFC components, multiple sclerosis functional composite including (25ft timed walk; 9-hole peg test (9-HPT), paced auditory serial addition test (PASAT)); FSS, fatigue severity scale; FLS, flu-like symptoms; QoL, quality of life; T2 BOD, T2 burden of disease; T25FW, timed 25 foot walk; TTW10, timed 10 foot tandem walk; BMI, body mass index; y, years; f, female; m, male; RCT, randomised controlled trial; SD, standard deviation; MS, multiple sclerosis. RRMS, relapsing-remitting multiple sclerosis; 25(OH)D, 25-hydroxy vitamin D.

**Table 3:** Inclusion and exclusion criteria of reviewed studies.

Study	Age	MS Diagnosis	EDSS Score	Serum 25(OH)D Level	Other Inclusion Criteria	Exclusion Criteria
[19]	18–50 years	MS	≤4.5	n/a	n/a	Inability to walk 500 m or more; conditions or medication affecting bone health; pregnancy, lactating during the past 6 months; menopause; unwillingness to use contraception
[21]	15–60 years	RRMS	≤6	>40 ng/mL	RRMS for 1–12 years, no relapse for at least one month; continue current medications	SPMS and PPMS; other conditions; use of vitamin D supplements; pregnancy
[12]	≥18 years	RRMS	<7	<75 nmol/L or (<30 ng/mL)	IFN-β therapy or those who continue to suffer from FLS beyond 4 months of treatment with IFN-β	Abnormalities of vitamin D related hormonal system; use of medications that influence vitamin D metabolism; conditions of increased susceptibility to hypercalcemia; pregnancy
[14]	18–55 years	RRMS	<5	<85 nmol/L or (<34 ng/mL)	IFN-β therapy for at least 1month and no neutralizing antibodies; contraception; at least one relapse during the year prior the study and/or MRI activity defined as presence of Gd-enhancing lesions on brain MRI	Serum calcium > 2.6 mmol/L; other conditions; pregnancy; use of other immunomodulatory therapy than INFB-1β; allergy to cholecalciferol or peanuts; alcohol or drug abuse
[16]	18–55 years	RRMS	<4	n/a	No relapse 30 days before inclusion; negative β-HCG test for women; calcium < 11 mg/dL	Pregnancy; lactation; other disease; receiving > 4000 IU of vitamin D, corticosteroids treatment in the previous 30 days; aspartate or alanine transaminase > 3xnormal values, ALP > 2.5xnormal values
[15]	18–50 years	RRMS	<4.5	n/a	n/a	Disease or medication affecting bone health; menopause; pregnancy; lactation; nephrolithiasis
[18]	18–55 years	RRMS	<4	<85 ng/mL	No relapse 30 days prior to study day; negative β-HCG test for women; calcium < 11 mg/dL; no relapse during the study	Pregnancy; lactation; other diseases; receiving >4000 IU of vitamin D, corticosteroids therapy in the previous 30 days; AST > 3xnormal values, ALP > 2.5xnormal values
[17]	18–50 years	RRMS	≤4	n/a	No relapse within 30 days prior to study day; first clinical event occurring within 5 years prior to screening; have had at least one relapse, or one or more Gd-enhancing or new T2 MRI lesions within the 12 months; receiving IFNβ-1a > 90 days and <12 months	Pregnancy or lactation; other diseases; use of corticosteroids or adrenocorticotrophic hormone within 30 days prior to SD1 abnormalities of vitamin D-related hormonal system; use of medications that influence vitamin D metabolism; taking N400 IU (N10 μg) of vitamin D supplement daily
[13]	18–55 years	RRMS	n/a	20–50 ng/mL	No relapse within 30 days; serum creatinine >1.5 mg/dL	Daily intake of vitamin D > 1000 IU or change of immunomodulatory therapy within the past 3 months, systemic glucocorticoid therapy; pregnancy, other condition
[20]	18–55 years	RRMS	≤5.0	<85 nmol/L	IFNB-1b use for at least 1month; no neutralising antibodies to IFNβ, as measured by the indirect myxovirus A (MxA) test, using appropriate contraceptive methods.	Pregnancy; serum calcium >2.6 mmol/L; primary hyperparathyroidism; alcohol or drug abuse; use of immunomodulatory therapy other than IFNB-1b; known allergy to cholecalciferol or peanuts; therapy with digitalis, calcitonin, vitamin D3 analogues or vitamin D; any condition predisposing to hypercalcaemia; significant hypertension (blood pressure < 180/110 mm Hg); hyperthyroidism or hypothyroidism in the year before the study began; a history of kidney stones in the previous 5 years; cardiac insufficiency or significant cardiac dysrhythmia; unstable ischaemic heart disease; depression; and inability to perform serial MRI scans.

MS, multiple sclerosis; RRMS, relapsing-remitting multiple sclerosis; EDSS, expanded disability status scale; FLS, flue like symptoms; HCG, Human chorionic gonadotropin; ALP, Alkaline phosphatase; AST, Aspartate transaminase; SPMS, secondary progressive multiple sclerosis; PPMS, primary progressive multiple sclerosis, SD1; study day 1; IFNβ, Interferon-β.

**Table 4:** Changes in serum 25(OH)D levels after intervention supplementation.

References	Within Group Differences	Between Group Differences
[19]	Intervention: 20,000 IU of vitamin D significantly increased serum 25(OH)D levels from a mean of 55.56 to 123.17 nmol/L. Control: there was only a minor increase from 57.33 to 61.80 nmol/L.	Significant difference in serum levels of 25(OH)D after 96 weeks between the intervention and control groups (*p* < 0.001).
[21]	n/a	n/a
[12]	Intervention: serum 25(OH)D levels significantly increased in a high-dose (4370 IU/d) groups from a mean of 48.2 to 122.6 nmol/L Control: low-dose (800 IU/d) from 48 to 68 nmol/L.	Significantly higher serum 25(OH-D) levels were reported in high dose group compared to low-dose arm after 48 weeks (*p* < 0.001).
[14]	Intervention: serum 25(OH)D levels increased significantly from a mean of 54 to 109 nmol/L. Placebo: decreased from a mean of 55 to 51 nmol/L after 48 weeks.	n/a
[16]	Intervention: Serum 25(OH)D levels rose from a median of 28.27 to 84.67 nmol/L. Placebo: fell from 39.6 to 28.66 nmol/L.	A significant difference after 12 weeks between groups (*p* < 0.001).
[15]	Intervention: serum levels of 25(OH)D significantly increased from 56 to 123nmol/L. Placebo: levels slightly increased from 57 to 63 nmol/L.	A significant difference in serum levels after 96 weeks between groups (*p* < 0.001).
[18]	Intervention: serum 25(OH)D levels significantly increased from a median of 28.27 to 84.67 ng/mL. Placebo: a decrease from 39.6 to 28.66 ng/mL.	These differences were significant between groups after 12 weeks (*p* < 0.001).
[17]	Intervention: serum 25(OH)D concentration increased significantly in the vitamin D group from 60 to 231 nmol/L. Placebo: changed to a lesser degree (54 to 60 nmol/L).	The was a significant difference after 48 weeks between the groups (*p* < 0.001).
[13]	High dose: Mean change of 34.9 ng/mL. Low dose: mean change of 6.9 ng/mL	A high dose of vitamin D resulted in significantly higher serum 25(OH-D) levels versus low-dose after 24 weeks (*p* < 0.00001).
[20]	Intervention: serum 25(OH)D levels increased from a mean of 54 to 110 nmol/L. Placebo: decreased from a mean of 56 to 50 nmol/L after 48 weeks	A significant difference between groups (*p* < 0.001).

**Table 5:** Key findings of reviewed studies.

Reference	Key findings	Significance	Conclusion
[19]	1. Serum 25(OH)D level significantly increased in intervention group vs control	1. *p* < 0.001	Supplementation did not result in beneficial effects on the measured MS-related outcomes; no significant difference between groups in ARR, EDSS, MSFC components, grip strength or fatigue
	2. ARR increased in intervention group vs control	2. *p* = 0.25	
	3. EDSS decreased in intervention group vs control	3. *p* = 0.97	
	4. MSFC components: 25ft timed walk decreased in intervention group vs control; 9-HPT increased in intervention group vs control; PASAT increased in intervention vs control;	4. *p* = 0.87; *p* = 0.35; *p* = 0.21	
	5. Grip strength decreased in intervention group vs control;	5. *p* = 0.76	
	6. Fatigue increased in intervention group vs control	6. *p* = 0.9	
[21]	1. Relapse rate significantly decreased in intervention and control groups; no significant difference in relapse rate between the groups;	1. *p* < 0.001; *p* < 0.001; *p* > 0.05;	No significant differences in the EDSS score or relapse rate between the vitamin D and control groups at the end of the study period; vitamin D supplementation at the doses used seems safe
	2. EDSS unchanged in intervention group and increased in control	2. N/A; *p* < 0.01	
[12]	1. Serum 25(OH)D levels increased in HDVD group vs LDVD group;	1. *p* < 0.001	Vitamin D supplementation was associated with dose-dependent changes in IL-17 serum levels, while not affecting IFN−β related FLS; vitamin D supplementation at the doses used seems safe
	2. PTH decreased in HDVD group but no significant change with LDVD;	2. *p* = 0.04; *p* = 0.17	
	3. No change in FLS	3. N/A	
	4. IL-17 levels increased in HDVD and LDVD groups;	4. *p* = 0.75; *p* = 0.04	
	5. No significant differences in relapse rate, EDSS, QoL, serum IL-10 and IFNγ;	5. *p* > 0.05	
	6. Serum calcium levels remained stable and within normal range in both dosage groups	6. *p* = 0.2; *p* = 0.4	
[14]	1. Serum levels of 25(OH)D increased in intervention group and edcreased in control;	1. N/A	Serum LAP (TGF-β) levels increased significantly in people receiving vitamin D; Therefore vitamin D might be useful in improving MRI outcomes; The levels of the other cytokines did not change significantly in either group
	2. Serum levels of LAP (TGF-β) increased in intervention and control group;	2. *p* = 0.0249; *p* = 0.173	
	3. The levels of serum IFN-gamma; IL-17A and in IL-9 increased in intervention group	3. *p* = 0.0519; *p* = 0.0666; *p* = 0.0679	
[16]	1. Serum 25(OH)D levels increased in intervention group;	1. *p* < 0.001	25(OH)D levels increased significantly in those treated with vitamin D; IL-10 level increased significantly in the intervention group and its anti-inflammatory effect may play a role in improving outcomes in MS
	2. IL-10 levels increased in intervention group;	2. *p* = 0.015	
	3. No significant differences in serum calcium between groups at baseline or after 3 months	3. *p* = 0.980; *p* = 0.302	
[15]	1. Serum 25(OH)D level increased in intervention versus control;	1. *p* < 0.001	25(OH)D levels increased significantly in vitamin D group versus control; No significant differences for any inflammation markers between groups
	2. The inflammation marker averages did not differ significantly between groups	2. *p* > 0.05	
[18]	1. Serum 25(OH)D level increased in intervention versus control group;	1. *p* < 0.001	25(OH)D levels increased significantly in people in the intervention group; Significant difference in EDSS between groups; No difference in IL-17 levels between vitamin D and control group
	2. EDSS scores differ between groups;	2. *p* = 0.033	
	3. Serum levels of IL-17 changed in intervention group;	3. *p* = 0.002	
	4. No significant differences in serum calcium between groups at baseline and after 12 weeks	4. *p* = 0.980; *p* = 0.302	
[17]	1. Serum 25(OH)D level increased in intervention versus control group;	1. *p* < 0.001	25(OH)D levels increased significantly in the vitamin D group; Supplementation of vitamin D did not result in a relative increase in the total amount of lymphocytes
	2. The total amount of lymphocytes are similar between baseline and week 48;	2. *p* > 0.05	
	3. The proportion of cells in the immune regulatory cell compartment (nTreg, iTreg and Breg) did not change in either group;	3. *p* > 0.05	
	4. IL4+ Th cells decreased in the control but not the intervention group;	4. *p* = 0.04; *p* = 0.92	
	5. T cell cytokine secretion increased (IL5, LAP (TGF-β)) in the control but not the intervention group	5. *p* = 0.02; *p* < 0.001 *p* = 0.06; *p* < 0.01	
[13]	1. Serum 25(OH)D level increased in HDVD group vs LDVD;	1. *p* < 0.00001	25(OH)D levels increased significantly in the vitamin D group; Vitamin D supplementation exhibited immunomodulatory effects including reduction of interleukin-17 and decreased the proportion of effector memory CD4+ T cells with concomitant increase in central memory CD4+ T cells and naive CD4+ T cells; 10,400 IU daily is safe and tolerable
	2. The proportion of interleukin-17+CD4+ T cells, CD161+CD4+ T cells, and effector memory CD4+ T cells, the proportion of central memory CD4+ T cells and naive CD4+ T cells increased in HDVD group	2. *p* = 0.016; *p* = 0.03; *p* = 0.021; *p* = 0.018; *p* = 0.04	
[20]	1. Serum 25(OH)D level significantly increased in intervention group vs control;	1. *p* < 0.001	Vitamin D3 add on treatment to IFNB reduces MRI T1 enhancing lesions. Vitamin D supplementation at the doses used seems safe.
	2. T2 BOD reduced in intervention group vs control;	2. *p* = 0.105	
	3. Total number of Gd enhancing T1 lesions significantly decreased in the intervention group vs control;	3. *p* = 0.004	
	4. Fewer new/enlarging T2 lesions in the intervention group vs control;	4. *p* = 0.286	
	5. Gd enhancing lesion volume decreased in intervention group vs control;	5. *p* = 0.320	
	6. MRI activity lower in intervention group vs control;	6. *p* = 0.322	
	7. ARR decreased in intervention group vs control;	7. N/A	
	8. EDSS decreased in intervention group vs control;	8. *p* = 0.071	
	9. TTW10 decreased in intervention group vs control;	9. *p* = 0.076	
	10. T25FW decreased in intervention group vs control	10. *p* = 0.932	

VD, vitamin D group; HDVD, high-dose vitamin D group; LDVD, low-dose vitamin D group; ARR, annualised relapse rate; EDSS, Expanded Disability Status Scale- scores range from 0 to 10; MSFC, MS functional composite including (25ft timed walk; 9-hole peg test (9-HPT), paced auditory serial addition test (PASAT)); FSS, fatigue severity scale- scores range from 1 (no fatigue) to 7; QoL, quality of life; FLS, flu-like symptoms; LAP, latency activated peptide, IFNβ, Interferon-β;.T2 BOD T25FW TTW10. T2 BOD, T2 burden of disease (BOD) on MRI scans; T25FW, timed 25 foot walk, TTW10, timed 10 foot tandem walk.

**Table 6:** Dose of vitamin D and concomitant immunomodulatory therapy used in selected studies.

Study	High-Dose of Vitamin D	Low-Dose of Vitamin D	Placebo	Concomitant Immunomodulatory Therapy and Vitamin D/Calcium Supplements
[19]	20,000 IU of vitamin D3 per week	✗	✔	500 mg/d calcium; no restrictions on vitamin D supplements
[21]	0.25 μg/d of calcitriol for 2 weeks and then 0.5 μg/d	✗	✔	IFNβ (86.0% of participants), statins (10.0%), or immunosuppressive drugs (4.0%)
[12]	4370 IU/d of vitamin D3	800 IU/d of vitamin D3	✗	IFNβ
[14]	20,000 IU of vitamin D3 per week	✗	✔	IFNβ
[16]	50,000 IU of vitamin D3 every 5days	✗	✔	IFNβ; interferon-β; participants were not allowed to take any other vitamin D supplements;
[15]	20,000 IU vitamin D3 per week	✗	✔	calcium supplementation (500 mg/d); no restrictions on regular vitamin D supplementation or immunomodulatory treatment (i.e., IFN-b, glatiramer acetate, or natalizumab)
[18]	50,000 IU of vitamin D3 every 5days	✗	✔	IFNβ
[17]	7000 IU/d of vitamin D3 for 4 weeks, followed by 14,000 IU/d of vitamin D3;	✗	✔	IFNβ-1a
[13]	10,400 IU/d of vitamin D3	400 IU/d of vitamin D3	✗	89% of participants received immunomodulatory therapy; multivitamin containing 400 IU of D3 and 1000 mg/d of calcium
[20]	20,000 IU of vitamin D3 per week	✗	✔	IFNβ-1b

IFNβ, Interferon-β.
